# Cancer treatment in determination of hearing loss^☆^^☆☆^

**DOI:** 10.1016/j.bjorl.2014.12.010

**Published:** 2015-10-17

**Authors:** Priscila Feliciano de Oliveira, Camila Silva Oliveira, Joice Santos Andrade, Tamara Figueiredo do Carmo Santos, Aline Cabral de Oliveira-Barreto

**Affiliations:** a Doctorade in Course at Graduate Program in Health Sciences, Universidade Federal de Sergipe (UFS), São Cristóvão, SE, Brazil; b Speech Pathology and Audiology Course, Universidade Federal de Sergipe (UFS), São Cristóvão, SE, Brazil; c Postdoctoral Fellow in Course at Department of Speech Pathology, Universidade Federal de São Paulo (UNIFESP), São Paulo, SP, Brazil

**Keywords:** Cancer, Hearing loss, Chemotherapy, Radiotherapy, Neoplasia, Perda auditiva, Quimioterapia, Radioterapia

## Abstract

**Introduction:**

Chemotherapy and radiotherapy in oncology have repercussions in hearing health, and can damage structures of the inner ear. These repercussions usually, result in a bilateral and irreversible hearing loss.

**Objective:**

To identify sensorineural hearing loss cases with complaints of tinnitus and difficulty in speech understanding and investigate their relationship with the types of chemotherapy and radiotherapy the patients received.

**Methods:**

Cross-sectional, clinical, observational, analytical, historical cohort study of 58 subjects treated in a public hospital in the state of Sergipe, diagnosed with neoplasia. The subjects were submitted to anamnesis, conventional pure tone audiometry, and speech recognition threshold.

**Results:**

Of the 116 ears, 25.9% presented sensorioneural hearing loss characterized by changes in high frequencies. There was a positive correlation between hearing loss and the association of chemotherapy and radiotherapy (*p* = 0.035; *R* = 0.196). The auditory complaint analysis shows that most of the subjects had tinnitus and speech understanding difficulty, even with a normal auditory threshold.

**Conclusions:**

Cancer treatment causes hearing loss, associated with the administration of chemotherapy and radiotherapy. Cyclophosphamide increased the risk of causing hearing loss. Complaints of tinnitus and speech understanding difficulty were observed.

## Resumo

**Introdução:**

O tratamento quimioterápico e radioterápico na oncologia tem repercussão na saúde auditiva e pode lesar estruturas da orelha interna. Ocasiona perda auditiva, geralmente bilateral e irreversível.

**Objetivo:**

Identificar casos de perda auditiva sensorioneural e sua relação com a média de sessões de quimioterapia e radioterapia, com queixas de zumbido e dificuldade de entendimento da fala, bem como sua relação com medicamentos quimioterápicos.

**Método:**

Estudo de coorte histórica com corte transversal, clínico, observacional, analítico e retrospectivo em 58 sujeitos de um hospital público de Sergipe diagnosticados com neoplasia. Realizou-se anamnese, avaliação audiológica tonal convencional e pesquisa do limiar de reconhecimento de fala.

**Resultados:**

Das 116 orelhas; 25,9% apresentaram perda auditiva sensorioneural caracterizada por alterações nas frequências agudas. Observou-se correlação significativa entre perda auditiva e associação da quimioterapia e radioterapia (p = 0,035; R = 0,196). Na análise das queixas auditivas, verificou-se que a maioria apresentou zumbido e dificuldade de entendimento de fala, mesmo com limiares auditivos normais.

**Conclusões:**

O tratamento oncológico gera perda auditiva, que foi determinada pela associação da quimioterapia e radioterapia. Ciclofosfamida aumentou as chances de gerar perda auditiva. Verificou-se presença de queixas de zumbido e dificuldade de entendimento da fala.

## Introduction

Currently, cancer is considered a public health problem with high prevalence. There is a projection of 27 million new cases for the year 2030 worldwide and 17 million deaths from the disease. In Brazil, estimates for the year 2014/2015 report approximately 580,000 new cases of the disease. Among the most prevalent forms, non-melanoma skin cancer, prostate, breast, colorectal, lung, and stomach cancers prevail.^1^

The basic modalities in cancer treatment involve surgery, chemotherapy, and radiotherapy. Currently, the combination of chemotherapy and radiotherapy has improved patient survival. However, none of these treatment modalities is free of side effects. Among their effects, hearing loss caused by ototoxicity, can be documented.^2^, ^3^, ^4^

In recent years, the number of studies reporting the influence of chemotherapeutic agents on hearing function has increased. The drugs employed belong to different classes, and some of them – aminoglycosides, antineoplastic agents, antibiotics, nonsteroidal anti-inflammatory drugs, diuretics and antihypertensives – are considered to be ototoxic drugs. Drugs included in the platinum group are the most devastating, generating auditory symptoms such as tinnitus and hearing sensitivity change.^2^, ^4^, ^5^ It has also been observed that vincristine, doxorubicin, gemcitabine, cyclophosphamide, oxaliplatin, and farmorubicin^6^ also are ototoxic.

Radiotherapy can also damage the auditory organ. This therapeutic modality promotes tumor cell destruction by its ionizing radiation beams. A pre-calculated dose of radiation is applied to tumor cells during a specified time period, in a volume of tissue that includes the tumor. Hearing loss is most commonly found in the treatment of head and neck tumors. Although there are few studies focused on the side effects of radiotherapy on hearing health, the literature shows a wide variation in the incidence of ototoxicity.^3^, ^7^

In both treatments, the higher concentration of toxic substances in the body is able to reach the organ of Corti and sensory epithelia of the posterior labyrinth, through the labyrinthine fluids. These substances compromise mainly the outer hair cells and can lead to cochlear symptoms; however, vestibular disorders may arise in a slow or insidious way, even after the end of treatment. Usually, hearing loss is bilateral, irreversible, associated with tinnitus and is high-frequency in audiometric configuration.^3^, ^5^, ^8^

Studies investigating hearing in oncologic patients using conventional audiometric frequencies, report variable symptoms. It is common to observe tinnitus, difficulty understanding conversation in noisy environments, and changes in speech discrimination.^9^

Studies in this area also report that the hearing loss caused by ototoxic substances is often underestimated. Even in the presence of a hearing disorder, patients only report hearing complaints in specific situations, such as in noisy environments. Other patients only exhibit partial understanding of a message, which makes it more difficult for relatives to detect the hearing loss.^10^

Studies on oncology and hearing health sought to promote early detection of hearing impairment and to implement preventive measures in order to improve the quality of life of this population. This research was specific; its objectives were to identify cases of sensorineural hearing loss and their relationship with chemotherapy and radiotherapy session means, and with tinnitus and speech understanding difficulty complaints, as well as their relation to the use of various chemotherapy drugs.

## Methods

This was a cross-sectional, clinical, observational, analytical, historical cohort study. All ethical guidelines were followed, and this project was approved by the Research Ethics Committee, under protocol No. 0066.0.107.000-11.

Data collection was conducted from March to November of 2013 in the physical space of the cancer ward of a public hospital in Sergipe. The sample comprised 58 subjects aged 25–59 years with pathological diagnosis of cancer who were undergoing chemotherapy and/or radiotherapy. The following drugs were utilized for chemotherapy: carboplatin, cisplatin, doxorubicin, epirubicin, farmorubicin, vincristine, actinomycin, gemcitabine, oxaliplatin, cyclophosphamide and fluorouracil.

Subjects were excluded from the study if they: were over 60 years of age (to avoid hearing loss by age); had a history of previous ear surgery; worked in a setting with noise exposure; had previous acoustic trauma; had received ototoxic medication prior to current treatment; had infections or congenital syndromes; exhibited outer ear canal obstruction and/or had previously received chemotherapy and/or radiotherapy. In addition, subjects with a diagnosis of head and neck cancer or conductive hearing loss were also excluded.

After signing the informed consent, the patients’ history was obtained and meatoscopy and conventional pure tone audiometry were performed, with a survey for speech reception threshold.

The anamnesis scheme was developed by the researchers, and consisted of information concerning the past history of the disease, treatment performed, drugs used, time of use of the drug, initiation of therapy, and overall hearing health.

The equipment used to perform pure tone audiometry and a speech reception threshold survey was the Interacoustics Ad Model 229 B audiometer with a TDH-39 headset in a soundproof booth that followed the equipment calibration standards proposed by the Brazilian Federal Council of Phonoaudiology on environmental noise measurement (resolution No. 365 of March 30, 2009). On examination of pure tone audiometry, hearing thresholds were surveyed by interoctave frequencies of 0.25–8 kHz by air conduction and of 0.5–4 kHz by bone conduction. The speech recognition threshold survey was conducted to confirm the tonal findings.

Thus, using the tonal audiometry findings, the sample was organized into two groups: Group 1, consisting of ears with normal hearing thresholds, and Group 2, ears with hearing loss.

Data were tabulated and processed by the software Statistical Package for Social Sciences (SPSS), version 17.0. For a description of data, tabular presentation of means, medians, standard deviations, maximum and minimum values, and percentages were used. Sample normality was verified using the Shapiro–Wilk test. To evaluate the relationship between variables, the Mann–Whitney and bivariate correlation (Spearman coefficient) tests were utilized. Values were considered significant at *p* ≤ 0.05 and the admitted *α* value was 0.1.

## Results

The sample consisted of 58 subjects (116 ears) with female predominance (93.1%), and mean age of 47.19 (±9.36) years. For Group 1, the mean age was 46.56 (±9.38), and for Group 2, 51.93 (±9.38) years.

The most frequent diagnosis was breast cancer (69%), followed by cervical (8.6%) and uterine (5.2%) cancers. Among the therapeutic procedures recommended by the medical staff, it was noted that 84.5% underwent surgery, and patients were also referred for chemotherapy and/or radiotherapy. Chemotherapy and radiotherapy usually occurred after surgery; 37.9% underwent chemotherapy, 15.5% radiotherapy, and 46.6% both chemotherapy and radiotherapy.

The number of chemotherapy sessions ranged from one to 36, with a mean of 7.56 sessions; while the number of radiotherapy sessions ranged from one to 70, with a mean of 18.31. By the time audiological evaluations were carried out, 1.7% of the population were starting treatment; 53.4% were in progress, and 44.8% had completed treatment.

The studied population used either a single chemotherapeutic agent or a combination of several drugs. The patients used the following drugs: carboplatin (*n* = 2), cisplatin (*n* = 6), doxorubicin (*n* = 6), epirubicin (*n* = 9), farmorubicin (*n* = 1), vincristine (*n* = 1) actinomycin (*n* = 1), gemcitabine (*n* = 6), and oxaliplatin (*n* = 3); the most frequently used agents were fluorouracil (*n* = 17) and cyclophosphamide (*n* = 18).

Of the 116 ears submitted for audiological evaluation, 30 (25.9%) exhibited sensorineural hearing loss, predominantly in the high frequencies. Audiometric analysis of the hearing loss cases revealed that worsening of tonal auditory thresholds began at the frequency of 4 kHz and gradually increased at the higher frequencies of 6 and 8 kHz (Table 1).

**Table 1:** Distribution of pure tone threshold means, by audiometric frequency test, in the population with normal audiograms and with sensorineural hearing loss (*n* = 116).

	Normal hearing thresholds (dBNA)	Sensorineural hearing loss (dBNA)
250 Hz	12.33	21.00
500 Hz	11.98	20.80
1000 Hz	10.52	25.40
2000 Hz	9.88	25.60
3000 Hz	10.35	25.40
4000 Hz	10.87	35.20
6000 Hz	13.66	39.80
8000 Hz	12.62	43.00

In cancer treatment, a positive bivariate correlation was found between hearing loss and the combination of chemotherapy and radiotherapy, with *p*-value = 0.035 and correlation coefficient *R* = 0.196. No significant relationship was observed for these treatments in isolation, as may be seen in Table 2, and the number of chemotherapy and radiotherapy sessions was not significant for determining the presence of hearing loss.

**Table 2:** Distribution of oncology treatment sessions in the studied population (n = 58).

	Group 1	Group 2	Mann–Whitney test^a^
Chemotherapy sessions, mean	7.93	6.44	*p* = 0.113
Radiotherapy sessions, mean	18.26	18.46	*p* = 0.616

a Significant *p*-value ≤ 0.05.

Regarding the type of chemotherapy drug used in the population presenting hearing loss, only cyclophosphamide showed a significant correlation (Table 3).

**Table 3:** *p*-Value calculated for the correlation between drug used in chemotherapy vs. presence of hearing loss (*n* = 30).

Drugs	*p*-Value^a^	*R*-value
Cyclophosphamide	0.034^b^	0.211
Carboplatin	0.66	–
Cisplatin	0.57	–
Doxorubicin	0.57	–
Epirubicin	0.89	–
Fluorouracil	1.00	–
Vincristine	0.39	–
Actinomycin	0.39	–
Gemcitabine	0.57	–
Oxaliplatin	0.18	–

*R*, correlation coefficient.

a Bivariate correlation test (Spearman's coefficient).

b Significant *p*-value ≤ 0.05.

With respect to self-reported hearing complaints by Group 1, 62.8% reported having a perception of tinnitus and 32.6% had difficulty listening; while in Group 2, 60.0% complained of tinnitus and 40.0% had difficulty listening. The presence of tinnitus was often observed, even without hearing loss (*p* = 0.79), and also occurred in conjunction with complaints of difficulty in understanding speech (*p* = 0.46).

Of the 15 subjects (30 ears) who experienced hearing loss, 23.3% reported worsening of hearing after chemotherapy and 6.7% after radiotherapy.

## Discussion

In this study, most participants were female, with prevalence of breast cancer, which confirm the findings in the literature for the state of Sergipe in 2014/2015.^1^

The population studied had a mean age over 40 years, similar to that found in previous studies.^11^ The subjects in Group 2 were older, compared to Group 1, and such data were also reported in the literature.^12^

Most respondents (84.5%) underwent surgery, and then were treated with chemotherapy and/or radiotherapy. The adopted medical management was also described in the literature, considering that, in the early staging process, the recommended treatment is surgery. This procedure enables a complete removal of the tumor and provides a better chance of survival. Subsequently, radiotherapy and chemotherapy are performed, independently or together.^13^

Most often, the medical team opted for a combination of chemotherapy and radiotherapy (46.6%), followed by chemotherapy (37.9%) and radiotherapy (15.5%). It is known that the type of treatment recommended is related to the type of tumor; and in the literature, high values for chemotherapy were found.^13^

With respect to the use of chemotherapeutic agents, it was found that cyclophosphamide was more significant in the determination of changes in auditory threshold; in the literature, this agent is referred to as ototoxic, possibly injuring cochlear hair cells.^14^

Hearing loss was mainly sensorineural, which is consistent with hearing loss caused by cochlear ototoxic substances, with early involvement of high frequencies.^2^, ^3^, ^11^, ^15^, ^16^

In the audiometric analysis, hearing threshold worsening occurred at and above the frequency of 4 kHz. Among the most affected frequencies, changes in 6–8 kHz have been diagnosed, which is in line with the literature.^17^ Researchers in the area report that the first frequencies to be affected are high (6 kHz and 8 kHz); and that during the patient's follow-up, hearing loss can advance to low frequencies. Nonetheless, other studies have been published reporting that the higher prevalence occurs at the frequencies of 3 kHz and 4 kHz.^11^

It was found that, of the 15 subjects with hearing loss, 23.3% reported worsening of post-chemotherapy hearing thresholds, and 6.7% reported after radiotherapy, with consequent hearing complaints. Studies have indicated that when there is change at the 4 kHz frequency, hearing complaints may occur. Thus, these data explain the hearing worsening in the population studied, since it was possible to detect hearing change from the frequency of 4 kHz onwards.^10^

The presence of tinnitus was considered as a relevant complaint. Tinnitus occurred in 62.8% of Group 1 and in 60.0% of Group 2. The literature also indicates a high incidence of tinnitus – a common occurrence in patients undergoing cancer treatment, which can be observed even in the absence of hearing loss.^2^, ^15^, ^17^, ^18^

There was no correlation between “difficulty in listening” and “suffering hearing loss.” This finding can be explained by the fact that the most affected frequencies are the highest ones, and the complaints are more frequent when they reach the speech range.^17^

With respect to cancer treatment, the means of radiotherapy and chemotherapy sessions were similar for both groups. The literature reports they need to consider that the greater the time of presence of toxic substances in the body, the greater the adverse effect, although there are individual differences in response to toxic agents, as well as variables that enable ototoxicity, e.g., family antecedents with deafness and individual susceptibility, among others.^17^, ^19^

## Conclusion

Hearing loss in subjects undergoing cancer treatment was observed, and the combination of chemotherapy and radiotherapy was instrumental for the change in hearing thresholds. Cyclophosphamide was the most frequently administered chemotherapeutic agent and was positively correlated with hearing loss. In the population submitted to cancer treatment, a high prevalence of tinnitus and speech understanding difficulty were found, even in the absence of hearing loss.

## Conflicts of interest

The authors declare no conflicts of interest.
